# Species Richness and Assemblages in Landscapes of Different Farming Intensity – Time to Revise Conservation Strategies?

**DOI:** 10.1371/journal.pone.0109816

**Published:** 2014-10-02

**Authors:** Erik Andersson, Regina Lindborg

**Affiliations:** 1 Stockholm Resilience Centre, Stockholm University, Stockholm, Sweden; 2 Dept. of Physical Geography and Quaternary Geology, Stockholm University, Stockholm, Sweden; Institute of Zoology, China

## Abstract

Worldwide conservation goals to protect biodiversity emphasize the need to rethink which objectives are most suitable for different landscapes. Comparing two different Swedish farming landscapes, we used survey data on birds and vascular plants to test whether landscapes with large, intensively managed farms had lower richness and diversity of the two taxa than landscapes with less intensively managed small farms, and if they differed in species composition. Landscapes with large intensively managed farms did not have lower richness than smaller low intensively managed farms. The landscape types were also similar in that they had few red listed species, normally targeted in conservation. Differences in species composition demonstrate that by having both types of agricultural landscapes regional diversity is increased, which is seldom captured in the objectives for agro-environmental policies. Thus we argue that focus on species richness or red listed species would miss the actual diversity found in the two landscape types. Biodiversity conservation, especially in production landscapes, would therefore benefit from a hierarchy of local to regional objectives with explicit targets in terms of which aspects of biodiversity to focus on.

## Introduction

Efforts to conserve biodiversity have traditionally focused mainly on natural habitats and pristine environments. During the last decades conservation focus has somewhat changed towards human dominated landscapes, especially farmlands [1], [2]. In regions like Europe and Asia, long traditions of low-intensive management have favoured biodiversity, and many species occurring in those landscapes are now dependent on human management for their survival (e.g. [1], [3], [4]). However, agricultural practices are changing and more modern landscapes of intensive food production often have little or no biodiversity left and in these landscapes biodiversity is mostly protected within national parks and nature reserves [5], [6]. This has resulted in polarized views on conservation management in agricultural landscapes, where land sparing separates intensively used agricultural land with high production from larger permanent preservation areas [6], [7] and land sharing incorporating small natural habitats within the agricultural system, often with lower yield per unit area. However, biodiversity conservation in regions with mixed land use intensities may need new strategies and targets that go species richness or red listed species.

The most common approach to survey biodiversity is still to collect binary data like presence/absence to get a measure of species richness [8]. This measure has been criticized for being crude and not capturing the differences between environments or the actual change when monitoring habitats over time as the link between species richness and e.g. phylogenetic diversity, functional diversity and community structure and identity of species is still controversial (e.g. [9]). Recent studies suggest that knowledge about species identity and community composition is especially important for maintaining ecosystem functions within a landscape [10], [11]. As a measure of the variation in species identity between sites, β diversity provides a link that connects diversity measures across scales, that is, between α (small scale) and γ (broad scale) diversities [12], [13]. Understanding the factors driving each of these components and their interrelationships provide useful insights for understanding the mechanisms that structure diversity and community composition in landscapes. The relationships between farming systems and biodiversity have so far mainly been studied at a very broad scale (e.g. [14]), focusing on the contrasting effects of the main farming systems, or at local scale investigating the relation between different crops and biodiversity (e.g. [15], [16]). As the most appropriate scale for investigating species movement and population dynamics most often is the intermediate landscape scale, studies on farming effects on biodiversity should be conducted at landscape scale where both the agricultural land and the surrounding matrix shape local biodiversity patterns [17]. This is also a relevant spatial scale for practical aspects of conservation biology since farms are the units for implementing agri-environmental policies.

This article examine the effect of two different Swedish landscapes types, one dominated by large-scale intensive farming and one by small-scale, low intensive farming, on richness, diversity, abundance and identity of bird and plant species. These two taxa can be expected to highlight different aspects of the same landscapes due to differences in the scales and environmental drivers they respond to. Specifically, we test the hypotheses that landscapes with intensively managed farms should have 1) lower richness, diversity and abundance of species, and 2) be different in species composition, i.e. have fewer rare and specialist species, than the low intensity, small scale farm landscapes. Further, based on our empirical findings, we discuss the broader issue of conservation objectives in relation to different dimensions of biodiversity and how different landscapes and scales may be more or less suited to different measures of and targets for biodiversity.

## Study Area

**Figure 1:**
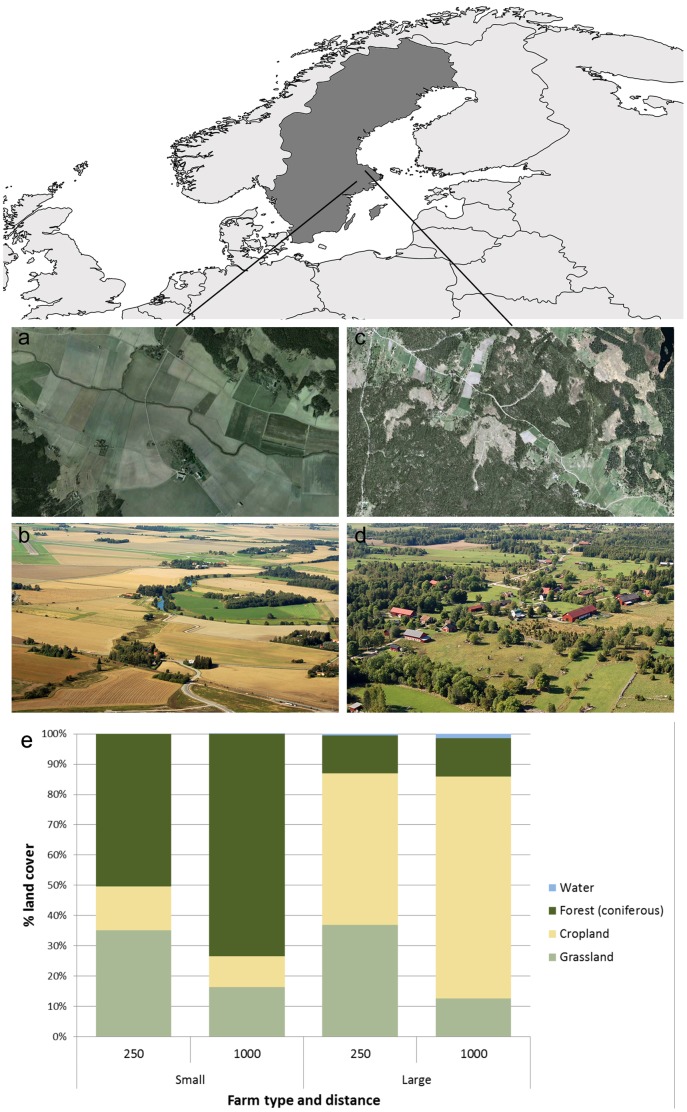
The two different landscape types. Pictures a) and b) illustrate the large farm landscapes and c) and d) the small farm landscapes. Picture e) shows the average land cover composition within 250 meters and 1000 meters, respectively, from each farmhouse in the different landscape types.

The study area is situated in south-central Sweden in the county of Uppsala (Fig. 1). In this area 16 farm-based sites were selected to represent the two landscape types; eight of the farms were large (mean size 336 ha; hereafter “large farms”), and found in an intensively managed agricultural area around Uppsala-Enköping-Västerås (59.680°N, 16.866°E); and eight were small scale and less intensively managed farms (on average 13 ha; hereafter “small farms”) located on the Hållnäs peninsula (60.553°N, 17.868°E; all farm locations can be found in Table S1 in Appendix S1). The farms were selected from County board data, where we chose the largest and smallest farms in the county, respectively. The county has homogeneous climate with July being the warmest month (average maximum temperature of 21°C), and January the coldest (with an average minimum of −8°C), with freezing spells that can last a number of consecutive days. Rainfall is higher during the summer months of the year (up to 60 mm/day), while less abundant in winter (up to 25 mm/day), accumulating around 530 mm at the end of the year. The two landscape types mainly differ in the relative proportion of agricultural land and forest, with near farmhouse environments being more similar in their composition than the larger surroundings (Fig. 1), and in the intensity of land use. As an effect of land use changes since the 1950s the landscapes types have developed along different trajectories. The landscapes surrounding the small farms have seen a trend of reforestation and a shift away from full-time family farming, although they still hold fragments of managed, semi-natural grasslands acknowledged for their high biodiversity. The large scale farming landscape has in turn seen a development towards larger farm units and intensified use.

However, due to different physical environment in the region, larger farms are always located in areas with richer, clay dominated soils and flatter topography, while small farms are mostly found in remote areas with poorer, sandy soils [18], [19]. This trend is evident all over Europe, where geophysical constrains like low soil fertility, steep slopes or stony grounds is a major limitation for mechanization and increased productivity (e.g. [20], [21]).

## Methods

Field observational data were collected at two levels of detail, strictly standardized (in space and time) abundance or frequency counts, and as presence - absence (standardized only in terms of time spent at a site) per site. The latter was used only for species lists. No legal permits were required for the data collection (Sweden has a general right to access also to private land). However, land owners were contacted before the surveys to obtain their informal approval.

### Bird survey

We used the point count method of surveying bird species and their abundances at each site [22]. Five survey points were located at and around each farmhouse. A central point was located next to the farmhouse and the other four in the NW, NE, SE and SW corners, respectively, 150 to 200 meters from the central point. With a mobile taxon like birds (e.g. [23]), this approach was deemed sufficient to capture the landscape variation and habitat diversity found at each site. Surveys were conducted two times: in early May and late May/early June 2012. We chose survey periods to coincide with the annual peak in singing activity. Surveys were begun at first light, at approximately 4:30am in early May and 3:30am late May/early June, and finished at latest 3 hours later. This period overlapped with the daily peak in bird vocal activity. Surveys were only conducted in mornings with favourable weather conditions, i.e. low winds and no heavy rain. At each point all birds seen or heard during five minutes and within two distance bands, 0–50 meters and 50–100 meters, were recorded, except birds that were detected flying over the counting station without landing. Birds that were farther away than 100 meters or seen or heard when walking between the points were recorded only as present, as were over-flying birds. In total, approximately 45 minutes were spent at each site per visit.

### Plant survey

Plant inventories for each farm were done in June-July 2011 and 2012 in four habitat types representative for Swedish rural landscapes and present in both landscapes, i.e. forest, semi-natural pasture, grazed ex-arable field and field margin, to compensate for plans being sessile. We selected habitat patches located adjacent (within a radius of 1 km) to the farmhouses. For each habitat and farm we inventoried presence/absence of vascular plants in10 randomly distributed 1 m^2^ plots, totalling 40 plots per farm. Based on this binary data we calculated the frequency of each plant species. In addition, a walking inventory of all species was also conducted [24] in one hectare of each habitat at each farm, noting all plants present but not found in the plots, spending approximately 20 minutes per habitat.

### Statistical analyses

Differences and/or similarities in community structure between the two types of landscapes were tested statistically using Bray-Curtis similarity and one-way analysis of similarities (ANOSIM) randomization test [25], a non-parametric analogue to the standard univariate 1- and 2-way ANOVA tests. Bray-Curtis dissimilarities were also used to estimate β-diversity and relative contribution of different species to these differences, the latter done through the SIMPER procedure [25]. Differences in species richness were tested with Welch’s t-test. As indicated above, each site has two data sets, one with species frequencies from the point/plot counts and a more inclusive species list including all species seen or heard at a site. The primary analyses were done on frequency data from the sample points/plots, complemented by analyses of the total species lists from each site. Frequency data were untransformed, using the relative abundances of different species, species lists were only presence-absence. Tests were done using PRIMER 6 software [26] and R version 3.01 (http://www.r-project.org/). To further analyse potential patterns we partitioned data into different classes based on species data such as red listed status [27] and primary habitat association. The habitats were: forest, open land, woodland, wetland and mixed/generalist for birds [28] and ruderal or grassland for plants [29]. Plant groups were chosen to reflect the hypothesis, i.e. capture the species normally in focus for conservation and species associated with poor environments. To test for spatial auto-correlation we used the RELATE test in the PRIMER 6 [26], a non-parametric form of Mantel test [30], usually employed to assess trends in time or space, but with good capacity to detect auto-correlation in a multivariate context. In this case, the Spearman rank matrix correlation (r) was computed between two resemblance matrices: one constructed as Bray-Curtis dissimilarities between the samples of species abundance and the other as (non-normalized) Euclidean distances calculated from the spatial coordinates (X and Y) determining the location of the 16 sites.

## Results

**Figure 2:**
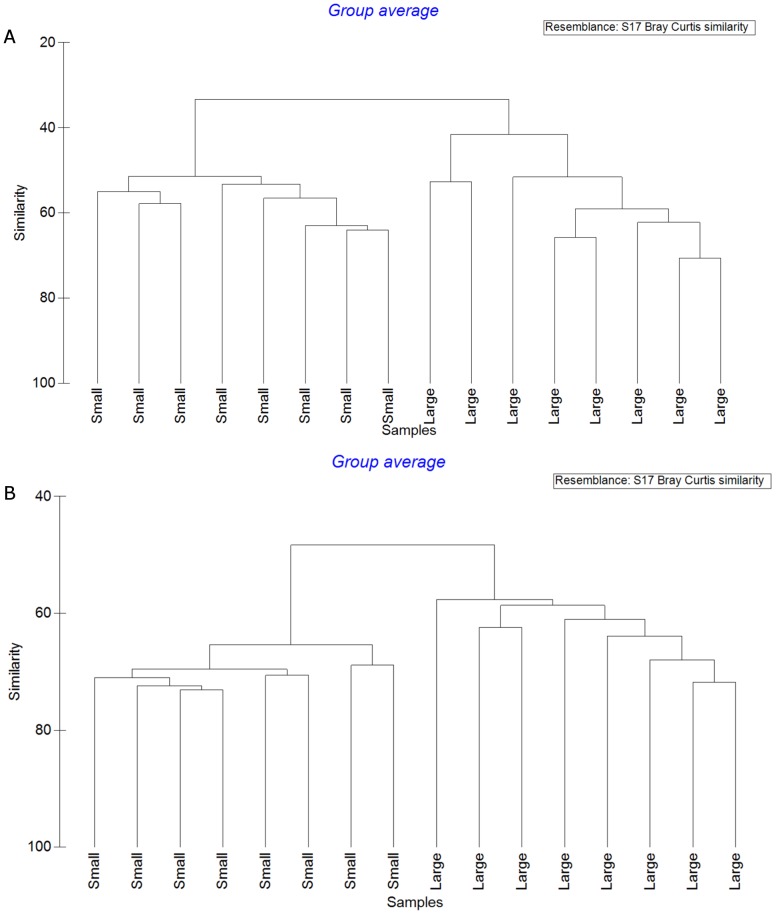
Within and between group similarities for a) birds and for b) plants in small and large farm landscapes. Similarities are based on Bray-Curtis dissimilarities.

In total we found 70 bird species, 55 in the small farm landscapes and 58 in the large, and 434 species of vascular plants, of which 316 species occurred in large farm landscapes and 300 species in small (Tables S2 & S3 in Appendix S1). Species richness was similar across farm types (Tables 1 & 2). However, bird and plant communities, respectively, showed statistically significant differences between large and small farm landscapes (Table 2 & Fig. 2), both in terms of species composition and abundance. Also the within group variation for birds and plants were different, with a higher similarity among small farm landscapes; ranging from 50% to 65% for birds and 70% to 75% for plants, compared to 50% to 70% for birds and 60% to 70% for plants on large farms (Fig. 2). Differences in bird communities were primarily caused by a smaller subset of species (seven species accounted for close to 50% of the difference between the landscape types, see Table 3). Plant communities did not have the same species driven differences (48 species to account for 50% of the difference). When analysed separately, two habitat guilds of birds demonstrated clear differences both in number of species and in species composition. Small scale farm landscapes had significantly more forest associated species while large farm landscapes had more species preferring open land. Plants did not signal such clear habitat related differences between the two farming landscapes; there were significantly more ruderal species in the large farm landscapes but we found no difference in the number of grassland plants (Table 4). However, both groups had significantly different species composition. Overall, the results showed that for both birds and plants the level of α-diversity was similar in all farms, whereas β-diversity was higher among the large farms (Fig. 2).

**Table 1:** Descriptive statistics for birds and plants based on a survey in large and small scale farming systems (n = 16).

		Mean species richness	STDEV	Shannon diversity (log e)	STDEV
**Birds**	Small scale, frequencies	24.5	±3.207	2.79	±0.17
	Large scale, frequencies	20.75	±4.334	2.57	±0.32
	Small scale, presence	29.25	±3.412	n/a	n/a
	Large scale, presence	28.375	±5.236	n/a	n/a
**Vascular plants**	Small scale, frequencies	107.875	±5.489	4.28	±0.054
	Large scale, frequencies	114.5	±6.459	4.36	±0.068
	Small scale, presence	170.125	±14.74	n/a	n/a
	Large scale, presence	177.125	±13.163	n/a	n/a

The table includes both survey plots (frequency) and overall (presence).

**Table 2:** Statistical significance of the differences in community compositions and species richness (within sample plots) between the two landscape types based on frequency data.

	ANOSIM, differences in community composition		t-test, differences in species richness		
	Sample statistics (Global R)	p- level	t	df	p-level
**Bird species**	0.775	0.001**	−1.967	12.897	0.071
**Plant species**	0.972	0.001**	1.002	13.825	0.334
**Ruderal plant species**	0.156	0.029*	2.966	11. 616	0.012*
**Grassland plant species**	0.694	0.001**	−0.222	13.792	0.828
**Birds associated with forest**	0.501	0.001**	−7.92	11.58	5.2e-6***
**Birds associated with open land**	0.79	0.001**	3.591	13.912	0.003**
**Birds associated with woodland**	0.04	0.237	0.5627	2.366	0.6224
**Generalist bird species**	0.126	0.066	−0.6333	9.939	0.5408
**Birds, other**	n/a	n/a	n/a	n/a	n/a

The ANOSIM analyses all used 999 permutations and the t-tests all had 1 degree of freedom. * indicates a p-level between 0.05 and>0.01, **between 0.01 and 0.001, and *** below 0.001.

**Table 3:** Bird species contributing the most to the difference between small and large farm landscapes, with 50% as the cut-off point for cumulative contribution.

Species	Av.Abund small farm	Av.Abund Large farm	Av.Diss	Diss/SD	Contrib%	Cum.%	Habitat association
Sky Lark	0.13	13.38	7.51	2.01	11.27	11.27	Open
Willow Warbler	11.13	1.63	5.41	1.89	8.12	19.39	Forest
Chaffinch	13.25	9.13	4.81	1.51	7.22	26.61	Forest/Woodland
House Sparrow & Tree Sparrow	0	7.38	4.1	1.39	6.15	32.76	Open
Great Tit	8	10.75	3.8	1.65	5.7	38.45	Forest/Woodland
Jackdaw	0.75	7.13	3.54	1.25	5.31	43.76	Open
Starling	1.5	6.63	3.17	1	4.76	48.52	Open

Dissimilarity measures are based on Bray-Curtis dissimilarities between communities.

**Table 4:** Descriptive statistics for different groups of vascular plants (ruderal, grassland, red listed species) and birds (forest, open and red listed birds) in large and small scale farming systems (n = 16).

	Classification	Landscape type	Mean number of species	Standard deviation
**Birds**	Forest	Small scale	12.375	±1.302
		Large scale	5.375	±2.134
	Open	Small scale	4.375	±2.387
		Large scale	8.5	±2.204
	Red listed	Small scale	0.75	±0.707
		Large scale	1.25	±0.707
**Plants**	Ruderal	Small scale	12.857	±1.345
		Large scale	15.625	±2.134
	Grassland	Small scale	34.375	±4.779
		Large scale	33.875	±4.224
	Red listed	Small scale	1.375	±1.506
		Large scale	1	±1.195

Red listed species were few and infrequent (Table 1 & 3), and while data were insufficient for statistical analysis, the actual species were not the same in the two landscape types (Tables S2 and S3 in Appendix S1). Among the red listed birds we found primarily species associated with open land, and two out of three of these were absent from the small scale farm landscapes. The small farm landscapes had one species associated with woodlands or mixed habitats but neither landscape type had any forest specialists. A majority of the red listed plants were associated to grassland habitats, and small farms held a few more species than large farms, but most often they occurred in both types of farms (Table S3 in Appendix S1).

The RELATE test show a potentially significant spatial effect in the comparison between the landscape types (p = 0.001 for birds and p = 0.001 for plants). However, it did not detect any spatial auto-correlation within the two landscape types (p = 0.522 and 0.216 for birds and p = 0.17 and 0.233 for plants, large:small).

## Discussion

Small farms with long traditional and extensive management have been shown to harbour higher species diversity compared to more intensively managed larger farms, not only as an effect of lower nutrient and pesticide input [31], but also due to higher landscape heterogeneity surrounding smaller farms (e.g. [7]). Therefore, we hypothesized that large farm landscapes should have lower richness, diversity and abundance of species and have fewer rare and specialist species than the small farm landscapes. Our results did not fully support these hypotheses. Although there were no statistical differences in the total number of species and number of red listed species the landscape types supported, the combined species lists for small and large farm landscapes, respectively, showed higher total number of birds and plants in the surroundings of the large farms. For plants, the landscape types were also similar in terms of the number of species associated with grasslands, which is surprising since grassland specialists are especially favoured by low intensive management [4], [19], [32]. Part of the explanation may be that the habitats where grassland specialists are found are managed in similar ways in the two landscape types. We did find more ruderal plant species in the large farm landscapes, which indirectly supports our hypothesis that these landscapes should have fewer specialists and rare species, i.e. the relative proportion rather than the absolute number of rare and specialized species was somewhat lower.

Given the similarities in species richness, it was somewhat surprising to find such big differences in community composition for both plants and birds, and in the identity of the few red listed species present. Although similar in α diversity, community differences indicate high β diversity between the two landscape types [33] (understood as the inverse of the community similarities presented in Fig. 2). Within the landscape types, β-diversity has been suggested to be higher in intensively managed agricultural landscapes, with larger distances between habitats [34], [35], a pattern in line with our findings. The more heterogeneous conditions could also explain the larger variation among bird communities. In addition, small scale farming has a longer, and perhaps more uniform, management tradition that might have acted as a stable environmental filter for community composition. The landscape composition at a regional scale, with large and small scale farming being separated in space, made it impossible to completely avoid spatial auto-correlation as a potential explanation for differences between the landscape types. However, we take the lack of spatial structuring within each landscape type as an indication that it is environmental differences rather than farm location that create the patterns we found.

For birds our richness estimates demonstrated clear differences between the landscape types in terms of the number of habitat specific species, differences that are congruent with regional differences in terms of the forest to open land ratio. The composition and land use of the larger surrounding landscape can be expected to influence organisms differently depending on their mobility, turnover time and resilience/persistence to change [36]. Thus, it was not unexpected that we found bird communities to reflect the larger landscape more clearly than plants. Mobile animals can benefit from complementary or supplementary resources available in the surrounding matrix outside the study site and hence be less confined by local resource limits (e.g. [37], [38]) compared to sessile organisms like plants [17]. Habitat specialists can be expected to have tolerance thresholds below which the amount of habitat is insufficient to attract them to a site [39], [40]. Although our study was not set up to identify neither the relevant spatial scale for different organisms nor the actual thresholds, our results indicate that despite being qualitatively similar (judging from the plant communities), open land in the small farm landscapes is clearly not extensive enough to attract many of the bird species associated with open land found in the large farm landscapes. Similarly, and more surprising since it does contain approximately 41% forest, the large farm landscapes are missing many of the forest associated species. Despite the relatively high proportion of forest the landscape may be less suited to support large populations due to non-optimal location in the landscape [41].

Placement of the semi-natural habitats within a landscape is crucial in the land sharing vs. land sparing debate. In our case, both small and large farm landscapes seem best described as land sharing since semi-natural habitats are patchy and dispersed. Locally based conservation strategies focusing on rare or threatened species seem less suited to the two landscape types and near farm environments in this study. Red listed species were few, and while a focus on them can be expected to support many other species and certain characteristics of the landscapes it would miss the much of the diversity actually there, not least on the regional scale. Land sharing, with habitats and resources more evenly distributed, may instead be well suited to ensure access to ecosystem services [42]. However, it may be that some land sharing landscapes are sinks for biodiversity and depend on a larger scale regional pool of species for their long term ability to provide ecosystem services [41], [43].

Ecosystem services are connected to biodiversity through functional traits, and thus related to community composition [11]. If biodiversity conservation would focus on habitats and community profiles our two taxa tell different stories, as is often the case [23], [44]. Bird communities, opposite to plants, clearly indicate that regional β-diversity is linked to large scale differences in the relative ratio between forest and open land, suggesting that large scale land use distributions would be the most suitable target for interventions and policies. Together, birds and plants show that conservation strategies must target several scales without compromising each other, and that the creation/maintenance of habitat alone will not necessarily predict or preserve differences or similarities between communities.

## Concluding Remark

Strategies targeting biodiversity conservation in agricultural landscapes should be couched differently, depending on the desired outcomes. If, as in our study, landscapes have relatively high species richness but few rare and specialist species, the most appropriate conservation measure is to focus on the more common species and how to secure ecological functions and the landscape-wide delivery of biodiversity related ecosystem services. This strategy corresponds best with a land sharing approach, i.e. maintaining small semi-natural biotopes interspersed in the landscape, which is also a good strategy for local biodiversity conservation. Furthermore, as we found the different landscapes to be similar in terms of α-diversity but different in their species communities, we argue that β-diversity should be a regional concern. At least for birds this means maintaining the different forest to open land ratios at a spatial scale larger than the farms. We argue that production landscapes should benefit from a nested hierarchy of scale specific conservation objectives, each with its explicit targets in terms of which aspects of biodiversity to focus on.

## Supporting Information

Appendix S1 **Location of all farms and all bird and plant species found in the inventory of small and large scale farms, related to habitat type and red list.** Table S1, Location of all 16 surveyed farms. Coordinates are given in GCS_SWEREF99, prime meridian is Greenwich and angular units are in in degrees. Table S2, All bird species found in the surveys, their red list status and habitat associations. Habitat associations are open, forest, woodland (scattered trees, otherwise not specific), water/forest (need both; not present in the point count), wetlands (including reeds) and generalist. Table S3, All vascular plant species found in the surveys, their red list status and whether they are associated with either ruderal land or traditional grasslands.
(DOCX)
