# Supplementary material for: Species Richness and Assemblages in Landscapes of Different Farming Intensity – Time to Revise Conservation Strategies?
Source: PLoS One. 2014 Oct 2;9(10):e109816. doi: 10.1371/journal.pone.0109816 (PMC4183564; doi:10.1371/journal.pone.0109816)
Supplement: Appendix S1 — Location of all farms and all bird and plant species found in the inventory of small and large scale farms, related to habitat type and red list. Table S1, Location of all 16 surveyed farms. Coordinates are given in GCS_SWEREF99, prime meridian is Greenwich and angular units are in in degrees. Table S2, All bird species found in the surveys, their red list status and habitat associations. Habitat associations are open, forest, woodland (scattered trees, otherwise not specific), water/forest (need both; not present in the point count), wetlands (including reeds) and generalist. Table S3, All vascular plant species found in the surveys, their red list status and whether they are associated with either ruderal land or traditional grasslands. (DOCX) [file pone.0109816.s001.docx]

Appendix S1. Location of all farms and all bird and plant species found in the inventory of small and large scale farms, related to habitat type and red list.

**Table S1**. Location of all 16 surveyed farms. Coordinates are given in GCS_SWEREF99, prime meridian is Greenwich and angular units are in in degrees.

| **Farm** | **Latitude** | **Longitude** |
| --- | --- | --- |
| Large #1 | 59.725240 | 17.393217 |
| Large #2 | 59.688640 | 16.887400 |
| Large #3 | 59.680921 | 16.863408 |
| Large #4 | 59.993110 | 17.558622 |
| Large #5 | 59.740650 | 17.181600 |
| Large #6 | 59.920118 | 17.602840 |
| Large #7 | 59.946609 | 17.820023 |
| Large #8 | 59.799767 | 17.374834 |
| Small #1 | 60.490689 | 17.870181 |
| Small #2 | 60.470776 | 17.877099 |
| Small #3 | 60.509708 | 17.920370 |
| Small #4 | 60.468570 | 17.974613 |
| Small #5 | 60.556752 | 17.942460 |
| Small #6 | 60.498727 | 17.913084 |
| Small #7 | 60.575000 | 17.944600 |
| Small #8 | 60.529200 | 17.863200 |

**Table S2**. All bird species found in the surveys, their red list status and habitat associations. Habitat associations are open, forest, woodland (scattered trees, otherwise not specific), water/forest (need both; not present in the point count), wetlands (including reeds) and generalist.

| **Bird species** | **Small farms** | **Large farms** | **Red list** | **Habitat** |
| --- | --- | --- | --- | --- |
| *Allauda arvensis* | x | x | x | Open |
| *Anas crecca* | x |  |  | Wetland |
| *Anas platyrhynchos* | x | x |  | Wetland |
| *Anser anser* |  | x |  | Open |
| *Anthus trivialis* | x | x |  | Forest |
| *Apus apus* | x | x |  | Open |
| *Buteo buteo* | x | x |  | Open |
| *Carduelis cannabina* |  | x | x | Open |
| *Carduelis carduelis* | x | x |  | Open |
| *Carduelis chloris* | x | x |  | Generalist |
| *Carduelis spinus* | x | x |  | Forest |
| *Certhia familiaris* | x | x |  | Woodland |
| *Columba oenas* |  | x |  | Forest |
| *Columba palumbus* | x | x |  | Forest |
| *Corvus corax* | x | x |  | Forest |
| *Corvus corone* | x | x |  | Generalist |
| *Corvus monedula* | x | x |  | Open |
| *Cuculus canorus* | x | x |  | Woodland |
| *Cygnus Cygnus* |  | x |  | Wetland |
| *Dendrocopus major* | x | x |  | Woodland |
| *Dendrocopus minor* |  | x |  | Forest |
| *Dryocopus martius* | x |  |  | Forest |
| *Emberiza citronella* | x | x |  | Open |
| *Emberiza schoeniclus* |  | x |  | Wetland |
| *Erithacus rubecula* | x | x |  | Forest |
| *Ficedula hypoleuca* | x | x |  | Forest |
| *Fringilla coelebs* | x | x |  | Generalist |
| *Gallinago gallinago* | x | x |  | Open |
| *Grus grus* | x | x |  | Forest |
| *Hippolais icterina* | x | x |  | Forest |
| *Hirundo rustica* | x | x |  | Open |
| *Jynx torquilla* | x |  | x | Generalist |
| *Lanius collurioides* |  | x |  | Open |
| *Loxia curvirostra* | x |  |  | Forest |
| *Luscinia luscinia* |  | x |  | Forest |
| *Motacilla alba* | x | x |  | Open |
| *Numenius arquata* |  | x | x | Open |
| *Oenanthe oenanthe* | x | x |  | Open |
| *Pandion haliaeetus* |  | x |  | Forest/water |
| *Parus ater* | x | x |  | Forest |
| *Parus caeruleus* | x | x |  | Generalist |
| *Parus crestatus* | x |  |  | Forest |
| *Parus major* | x | x |  | Generalist |
| *Passer domesticus & montanus* |  | x |  | Open |
| *Phasianus colchicus* |  | x |  | Open |
| *Phoenicurus phoenicurus* |  | x |  | Forest |
| *Phylloscopus collybita* | x |  |  | Woodland |
| *Phylloscopus sibilatrix* | x |  |  | Forest |
| *Phylloscopus trochilus* | x | x |  | Forest |
| *Phylloscopus trochilus* | x | x |  | Forest |
| *Pica pica* | x | x |  | Generalist |
| *Picus viridis* | x | x |  | Generalist |
| *Pluvialis apricaria* |  | x |  | Open |
| *Prunella modularis* | x |  |  | Forest |
| *Pyrrhula pyrrhula* | x |  |  | Forest |
| *Regulus regulus* | x | x |  | Forest |
| *Sitta europaea* | x | x |  | Woodland |
| *Sturnus vulgaris* | x | x |  | Open |
| *Sylvia atricapilla* | x | x |  | Forest |
| *Sylvia communis* | x | x |  | Open |
| *Sylvia curruca* | x | x |  | Open |
| *Tetrao tetrix* | x |  |  | Forest |
| *Tringa ochropus* | x |  |  | Forest |
| *Troglodytes troglodytes* | x | x |  | Forest |
| *Turdus iliacus* | x | x |  | Generalist |
| *Turdus merula* | x | x |  | Woodland |
| *Turdus philomenos* | x | x |  | Forest |
| *Turdus pilaris* | x | x |  | Generalist |
| *Turdus viscivorus* | x |  |  | Forest |
| *Vanellus vanellus* |  | x |  | Open |

**Table S3**. All vascular plant species found in the surveys, their red list status and whether they are associated with either ruderal land or traditional grasslands.

| **Plant species** | **Small farms** | **Large farms** | **Red list** | **Ruderal** | **Grassland species** |
| --- | --- | --- | --- | --- | --- |
| *Abies alba* | x | x |  |  |  |
| *Acer platanoides* | x | x |  |  |  |
| *Achillea millefolium* | x | x |  |  | x |
| *Achillea ptarmica* |  | x |  |  |  |
| *Actaea spicata* | x | x |  |  |  |
| *Aegopodium podagraria* |  | x |  |  |  |
| *Agrimonia eupatoria* | x | x |  |  | x |
| *Agrostis capillaris* |  | x |  |  | x |
| *Agrostis gigantea* |  | x |  |  |  |
| *Agrostis stolonifera* | x | x |  |  |  |
| *Ajuga pyramidalis* | x |  |  |  | x |
| *Alchemilla glaucescens* | x |  |  |  | x |
| *Alisma plantago-aquatica* | x | x |  |  |  |
| *Allium oleraceum* | x | x |  |  | x |
| *Allium x hollandicum* | x |  |  |  |  |
| *Alnus glutinosa* |  |  |  |  |  |
| *Alopecurus aequalis* | x | x |  |  |  |
| *Alopecurus geniculatus* | x | x |  |  |  |
| *Alopercurus pratensis* | x | x |  |  |  |
| *Anchusa arvensis* | x | x |  |  |  |
| *Anemone nemorosa* | x | x |  |  |  |
| *Angelica sylvestris* | x | x |  |  |  |
| *Antennaria dioica* |  |  |  |  | x |
| *Anthemis tinctoria* | x | x |  |  |  |
| *Anthoxanthum odoratum* | x | x |  |  | x |
| *Anthriscus sylvestris* |  | x |  | x |  |
| *Anthyllis vulneraria* | x |  |  |  | x |
| *Aquilegia vulgaris* | x | x |  |  |  |
| *Arabis glabra* | x | x |  |  |  |
| *Arctium minor* |  | x |  |  |  |
| *Arctium tomentosum* |  | x |  |  |  |
| *Arenaria serpyllifolia* |  | x |  |  | x |
| *Armoracia rusticana* |  | x |  |  |  |
| *Arrhenatherum elatius* |  | x |  | x |  |
| *Artemisia vulgaris* | x | x |  |  | x |
| *Athyrium filix-femina* |  | x |  |  |  |
| *Avena sativa* |  | x |  |  |  |
| *Barbarea vulgaris* | x | x |  |  |  |
| *Berberis vulgaris* | x | x |  |  |  |
| *Betula pendula* | x | x |  |  |  |
| *Betula pubescens* | x |  |  |  |  |
| *Bidens tripartita* | x | x |  | x |  |
| *Bistorta vivipara* | x |  |  |  | x |
| *Botrychium lunaria* | x |  | x |  | x |
| *Brachypodium pinnatum* | x |  |  |  |  |
| *Brachypodium sylvaticum* | x |  |  |  |  |
| *Briza media* |  | x |  |  | x |
| *Bromopsis inermis* |  |  |  |  | x |
| *Bromus hordeaceus* |  |  |  |  | x |
| *Bunias orientalis* | x | x |  |  |  |
| *Calamagrostis arundinacea* |  | x |  | x |  |
| *Calamagrostis canescens* | x | x |  |  |  |
| *Calluna vulgaris* | x | x |  |  |  |
| *Caltha palustris* | x |  |  |  |  |
| *Calystegia sepium* |  |  |  | x |  |
| *Camomilla suaveolens* | x | x |  |  |  |
| *Campanula glomerata* | x | x |  |  |  |
| *Campanula latifolia* | x | x |  |  |  |
| *Campanula patula* | x |  |  |  |  |
| *Campanula persicifolia* |  | x |  |  |  |
| *Campanula rapunculoides* | x | x |  |  |  |
| *Campanula rotundifolia* | x | x |  |  | x |
| *Capsella bursa-pastoris* | x | x |  | x |  |
| *Cardamine pratensis* | x |  |  |  | x |
| *Carduus crispus* |  | x |  |  |  |
| *Carex acuta* |  | x |  |  |  |
| *Carex canescens* | x | x |  |  |  |
| *Carex digitata* | x | x |  |  |  |
| *Carex disticha* | x |  |  |  |  |
| *Carex echinata* | x | x |  |  | x |
| *Carex elata* | x |  |  |  |  |
| *Carex elongata* | x |  |  |  |  |
| *Carex flacca* | x | x |  |  |  |
| *Carex flava* | x |  |  |  |  |
| *Carex hartmanii* | x |  | x |  |  |
| *Carex hirta* | x | x |  |  |  |
| *Carex hostiana* | x |  | x |  |  |
| *Carex nigra* | x | x |  |  |  |
| *Carex ovalis* | x | x |  |  |  |
| *Carex pallescens* | x | x |  |  | x |
| *Carex panicea* | x | x |  |  |  |
| *Carex pilulifera* | x | x | x |  | x |
| *Carex rostrata* |  | x |  |  |  |
| *Carex spicata* | x | x |  |  | x |
| *Carex vaginata* |  |  |  |  |  |
| *Carex vulpina* |  |  |  |  |  |
| *Carlina vulgaris* | x | x |  |  |  |
| *Carum carvi* |  | x |  | x |  |
| *Centaurea cyanus* | x | x |  |  |  |
| *Centaurea jacea* | x | x |  |  | x |
| *Centaurea scabiosa* | x | x |  |  |  |
| *Cerastium fontanum* | x | x |  |  | x |
| *Chelidonium majus* | x | x |  | x |  |
| *Chenopodium album* |  | x |  |  |  |
| *Chenopodium rubrum* |  | x |  |  |  |
| *Chenopodium suecicum* | x | x |  |  |  |
| *Cichorium intybus* | x |  |  |  |  |
| *Cirsium arvense* | x | x |  | x |  |
| *Cirsium helenioides* | x |  |  |  |  |
| *Cirsium palustre* | x | x |  |  |  |
| *Cirsium vulgare* |  | x |  | x |  |
| *Consolida regalis* | x | x | x |  |  |
| *Convallaria majalis* |  | x |  |  |  |
| *Convolvulus arvense* | x | x |  | x |  |
| *Corylus avellana* | x | x |  |  |  |
| *Cotoneaster integerrimus* |  | x |  |  | x |
| *Crataegus monogyna* | x | x |  |  |  |
| *Crepis paludosa* | x |  |  |  |  |
| *Crepis praemorsa* |  |  |  |  | x |
| *Crepis tectorum* | x | x |  |  |  |
| *Cystopteris fragilis* | x | x |  |  |  |
| *Dactylis glomerata* | x | x |  |  |  |
| *Dactylorhiza maculata* | x |  |  |  |  |
| *Dactylorhiza virides* | x |  |  |  |  |
| *Danthonia decumbens* | x |  |  |  | x |
| *Daphne mezereum* | x |  |  |  |  |
| *Deschampsia cespitosa* | x | x |  |  |  |
| *Deschampsia flexuosa* | x | x |  |  |  |
| *Dianthus deltoides* | x | x |  |  | x |
| *Dryopteris carthusia* | x | x |  |  |  |
| *Dryopteris filix-mas* |  | x |  | x |  |
| *Elymus caninus* | x | x |  | x |  |
| *Elytrigia repens* | x | x |  |  |  |
| *Epilobium angustifolium* |  | x |  | x |  |
| *Epilobium ciliatum* | x | x |  |  |  |
| *Epilobium montanum* | x | x |  |  |  |
| *Epipactis helleborine* | x |  |  |  |  |
| *Equisetum arvense* |  | x |  |  |  |
| *Equisetum fluviatile* | x | x |  |  |  |
| *Equisetum heymale* | x |  |  |  |  |
| *Equisetum palustre* |  |  |  |  |  |
| *Equisetum pratensis* | x |  |  | x |  |
| *Equisetum sylvaticum* | x |  |  |  |  |
| *Eriophorum angustifolium* | x |  |  |  |  |
| *Eriophorum gracile* | x |  |  |  |  |
| *Eriophorum vaginatum* |  |  |  |  |  |
| *Erodium cicutarium* | x |  |  |  |  |
| *Erysimum cheiranthoides* |  | x |  |  |  |
| *Euphorbia helioscopia* |  | x |  |  |  |
| *Euphrasia stricta* |  | x | x |  | x |
| *Fagus sylvatica* | x | x |  |  |  |
| *Fallopia convolvulus* |  | x |  |  |  |
| *Fallopia dumetorum* | x | x |  |  |  |
| *Festuca arundinacea* | x |  |  |  |  |
| *Festuca ovina* | x | x |  |  | x |
| *Festuca pratensis* |  | x |  |  |  |
| *Festuca rubra* | x | x |  |  | x |
| *Filipendula ulmaria* | x | x |  | x |  |
| *Filipendula vulgaris* | x | x |  |  | x |
| *Fragaria vesca* | x | x |  |  | x |
| *Frangula alnus* | x | x |  |  |  |
| *Fraxinus excelsior* | x | x |  |  |  |
| *Fumaria officinalis* | x | x |  |  |  |
| *Galeopsis bidifa* |  |  |  |  |  |
| *Galeopsis ladanum* |  | x | x |  |  |
| *Galeopsis speciosa* |  | x |  |  |  |
| *Galeopsis tetrahit* | x | x |  |  |  |
| *Galium album* |  | x |  |  | x |
| *Galium aparine* | x | x |  |  |  |
| *Galium boreale* | x | x |  |  |  |
| *Galium palustre* |  | x |  |  |  |
| *Galium spurium* | x | x |  |  |  |
| *Galium uliginosum* | x | x |  |  | x |
| *Galium verum* |  | x |  |  | x |
| *Gentianella campestris* | x |  | x |  | x |
| *Gentianella campestris* |  |  | x |  | x |
| *Geranium pratense* |  | x |  |  |  |
| *Geranium pusillum* | x | x |  | x |  |
| *Geranium robertianium* | x | x |  |  |  |
| *Geranium sanguineum* | x |  |  |  |  |
| *Geranium sylvaticum* | x | x |  | x |  |
| *Geum rivale* | x | x |  |  |  |
| *Geum urbanum* | x | x |  |  |  |
| *Glechoma hederacea* | x | x |  |  |  |
| *Glyceria fluitans* | x | x |  |  |  |
| *Glyceria maxima* |  |  |  |  |  |
| *Gnaphalium sylvaticum* | x |  |  |  | x |
| *Gnaphalium uliginosum* | x | x |  |  |  |
| *Gymnadenia conopsea* | x |  |  |  |  |
| *Gymnocarpium dryopteris* | x | x |  |  |  |
| *Helianthemun nummularium* | x | x |  |  | x |
| *Helictotrichon pratense* | x | x |  |  |  |
| *Helictotrichon pubescens* | x | x |  |  |  |
| *Hepatica nobilis* | x | x |  |  |  |
| *Heracleum sphondylium* |  | x |  |  |  |
| *Hesperis matronalis* | x | x |  |  |  |
| *Hieracium sect. Hieracium* | x | x |  |  |  |
| *Hieracium sect. Vulgata* | x | x |  |  | x |
| *Hieracium umbellatum* | x | x |  |  |  |
| *Hierochloë odorata* | x |  |  |  |  |
| *Hippophaë rhamnoides* | x |  |  |  |  |
| *Hypericum maculatum* |  | x |  |  |  |
| *Hypericum perforatum* | x | x |  |  | x |
| *Hypochoeris maculata* |  |  |  |  |  |
| *Inula salicina* | x | x |  |  | x |
| *Iris pseudacorus* |  |  |  |  |  |
| *Juncus alpinoarticulatus* | x |  |  |  |  |
| *Juncus articulatus* | x |  |  |  |  |
| *Juncus bufonius* | x |  |  |  |  |
| *Juncus compressus* | x |  |  |  |  |
| *Juncus conglomeratus* |  | x |  |  |  |
| *Juncus effusus* | x | x |  |  |  |
| *Juncus filiformis* | x |  |  |  |  |
| *Juniperus communis* | x | x |  |  |  |
| *Knautia arvensis* |  | x |  |  |  |
| *Lactuca serriola* | x | x |  |  |  |
| *Lamium hybridum* |  | x |  |  |  |
| *Lamium hybridum* |  | x |  |  |  |
| *Lamium purpureum* |  | x |  |  |  |
| *Lamium sp.* | x | x |  |  |  |
| *Lapsana communis* | x | x |  |  |  |
| *Laserpitium latifolium* |  |  |  |  |  |
| *Lathyrus linifolius* | x | x |  |  |  |
| *Lathyrus pratensis* | x | x |  |  | x |
| *Lathyrus vernus* | x |  |  |  | x |
| *Leontodon autumnalis* | x | x |  |  | x |
| *Leucanthemum vulgare* | x | x |  |  | x |
| *Linnaea borealis* | x |  |  |  | x |
| *Linum catharticum* | x |  |  |  |  |
| *Listera ovata* |  |  |  |  | x |
| *Lolium perenne* |  | x |  |  |  |
| *Lonicera periclymenum* | x | x |  | x |  |
| *Lonicera xylosteum* | x | x |  |  |  |
| *Lotus corniculatus* |  | x |  |  |  |
| *Luzula campestris* | x | x |  |  | x |
| *Luzula multiflora* | x | x |  |  | x |
| *Luzula pallescens* | x |  |  |  |  |
| *Luzula pilosa* |  | x |  |  |  |
| *Lychnis flos-cuculi* | x |  |  |  |  |
| *Lychnis viscaria* | x |  |  |  |  |
| *Lycopodium clavatum* | x |  |  |  | x |
| *Lysimachia thyrsiflora* | x |  |  |  |  |
| *Lysimachia vulgaris* | x |  |  |  |  |
| *Maianthemum bifolium* | x | x |  | x |  |
| *Malus domestica* | x | x |  |  |  |
| *Malus sylvestris* |  |  |  |  |  |
| *Malva moschata* | x | x |  |  |  |
| *Matricaria matricarioides* | x |  |  |  |  |
| *Medicago lupulina* |  | x |  |  |  |
| *Medicago sativa* |  | x |  |  | x |
| *Medicago sativa* | x | x |  |  |  |
| *Melampyrum nemorosum* | x |  |  |  |  |
| *Melampyrum pratense* | x |  |  |  |  |
| *Melampyrum sylvaticum* | x | x |  |  |  |
| *Melica nutans* | x | x |  |  |  |
| *Mentha arvensis* |  | x |  |  |  |
| *Mentha x verticillata* | x |  |  |  |  |
| *Milium effusum* |  | x |  |  |  |
| *Moehringia trinervia* | x | x |  |  |  |
| *Molinia caerulea* |  |  |  |  |  |
| *Monotropa hypopitys* | x |  |  |  |  |
| *Mycelis murialis* | x | x |  |  |  |
| *Myosotis arvensis* | x | x |  |  |  |
| *Myosotis laxa* |  | x |  | x |  |
| *Myosotis ramossisima* |  | x |  |  |  |
| *Myosotis sylvatica* | x | x |  |  | x |
| *Myrica gale* | x |  |  |  |  |
| *Nardus stricta* | x |  |  |  |  |
| *Neottia nidus-avis* |  |  |  |  | x |
| *Origanum vulgare* | x | x |  |  |  |
| *Oxalis acetosella* |  | x |  |  |  |
| *Papaver dubium* | x | x |  |  |  |
| *Paris quadrifolia* |  | x |  |  |  |
| *Persicaria hydropiper* | x |  |  |  |  |
| *Persicaria lapathifolia* |  | x |  |  |  |
| *Persicaria maculosa* |  | x |  |  |  |
| *Phagmites australis* | x |  |  |  | x |
| *Phalaris arundinacea* | x |  |  | x |  |
| *Phleum phleoides* |  | x |  |  |  |
| *Phleum pratense* | x | x |  |  |  |
| *Phleum pratense* | x |  |  |  |  |
| *Picea abies* | x | x |  |  |  |
| *Pilosella lactucella* | x | x |  |  | x |
| *Pilosella officinarum* | x | x |  |  |  |
| *Pimpinella saxifraga* | x | x |  |  | x |
| *Pinus sylvestris* | x | x |  |  |  |
| *Plantago lanceolata* | x | x |  |  | x |
| *Plantago major* |  | x |  | x |  |
| *Plantago media* | x | x |  |  | x |
| *Platanthera bifolia* | x |  |  |  | x |
| *Platanthera chlorantha* | x | x |  |  |  |
| *Poa annua* |  | x |  | x |  |
| *Poa compressa* | x | x |  |  |  |
| *Poa nemoralis* | x | x |  |  |  |
| *Poa pratensis* |  | x |  | x |  |
| *Poa trivialis* | x | x |  |  |  |
| *Polygala vulgaris* | x | x |  | x |  |
| *Polygonatum odoratum* | x | x |  |  |  |
| *Polygonum aviculare* | x | x |  | x |  |
| *Polypodium vulgare* | x | x |  |  |  |
| *Populus tremula* | x | x |  |  |  |
| *Potentilla anserina* | x | x |  | x |  |
| *Potentilla argentea* | x | x |  |  | x |
| *Potentilla erecta* | x | x |  |  |  |
| *Potentilla palustris* | x |  |  |  |  |
| *Potentilla reptans* |  | x |  |  | x |
| *Primula farinosa* | x |  | x |  | x |
| *Primula veris* | x | x |  |  | x |
| *Prunella vulgaris* | x | x |  |  |  |
| *Prunus avium* | x | x |  |  |  |
| *Prunus domestica* | x |  |  |  |  |
| *Prunus padus* | x | x |  |  |  |
| *Prunus spinosa* | x | x |  |  |  |
| *Pteridium aquilinum* |  | x |  |  |  |
| *Pyrola chloranta* | x | x |  |  |  |
| *Pyrola rotundifolia* | x |  |  |  |  |
| *Quercus robur* | x | x |  |  |  |
| *Ranunculus acris* | x | x |  |  |  |
| *Ranunculus auricomus* | x | x |  |  | x |
| *Ranunculus bulbosus* | x |  |  |  | x |
| *Ranunculus flammula* | x | x |  |  |  |
| *Ranunculus polyanthemos* | x | x |  |  | x |
| *Ranunculus repens* |  | x |  |  |  |
| *Ranunculus sceleratus* |  | x |  |  |  |
| *Rhamnus cathartica* | x | x |  |  |  |
| *Rhinanthus minor* | x | x |  |  | x |
| *Ribes alpinum* | x |  |  |  |  |
| *Ribes nigrum* | x |  |  |  |  |
| *Ribes rubrum* | x |  |  |  |  |
| *Ribes spicatum* |  |  |  |  |  |
| *Ribes uva-crispa* |  | x |  |  |  |
| *Rosa canina* | x | x |  |  |  |
| *Rosa dumalis* | x | x |  |  |  |
| *Rosa sherardi* | x | x |  |  |  |
| *Rosa villosa* | x | x |  |  |  |
| *Rubus idaeus* | x | x |  |  |  |
| *Rubus saxatilis* | x | x |  |  |  |
| *Rumex acetosa* | x | x |  |  | x |
| *Rumex acetosella* | x | x |  |  | x |
| *Rumex crispus* | x | x |  |  |  |
| *Rumex longifolius* |  | x |  |  |  |
| *Rumex obtusifolius* | x |  |  | x |  |
| *Sagina procumbens* | x |  |  |  |  |
| *Salix caprea* | x | x |  |  |  |
| *Salix cinerea* | x | x |  |  |  |
| *Salix myrsinifolia s* | x |  |  |  |  |
| *Salix pentandra* | x |  |  |  |  |
| *Salix repens* |  | x |  |  |  |
| *Sambucus racemosa* | x | x |  |  |  |
| *Sanicula europaea* |  |  |  |  |  |
| *Satureja acinos* | x | x |  |  | x |
| *Satureja vulgaris* | x |  |  |  |  |
| *Saxifraga granulata* |  |  |  |  | x |
| *Scleranthus annuus* |  | x |  |  | x |
| *Scleranthus perennis* |  | x |  |  |  |
| *Scorzonera humilis* | x | x |  |  | x |
| *Scrophularia nodosa* | x | x |  |  |  |
| *Sedum acre* |  | x |  |  | x |
| *Sedum sexangulare* | x | x |  |  |  |
| *Sedum telephium* |  | x |  |  |  |
| *Selinum carvifolia* | x |  |  |  |  |
| *Senecio sylvaticus* |  | x |  |  |  |
| *Senecio viscosus* |  | x |  | x |  |
| *Senecio vulgare* | x | x |  | x |  |
| *Serratula tinctoria* | x |  | x |  | x |
| *Seseli libanotis* |  | x |  |  |  |
| *Sesleria caerulea* |  |  |  |  |  |
| *Silene dioca* |  |  |  |  |  |
| *Silene nutans* |  | x |  |  | x |
| *Sinapsis arvensis* |  | x |  |  |  |
| *Solanum dulcamara* | x | x |  |  |  |
| *Solidago virgaurea* | x | x |  |  |  |
| *Sonchus arvensis* | x | x |  |  |  |
| *Sonchus asper* |  | x |  |  |  |
| *Sonchus oleraceus* |  | x |  |  |  |
| *Sorbus aucuparia* | x | x |  |  |  |
| *Sorbus intermedia* | x | x |  |  |  |
| *Spiraea japonica* |  |  |  |  |  |
| *Spiraea sp.* | x | x |  |  |  |
| *Stachys byzantina* | x |  |  |  |  |
| *Stachys macrantha* |  | x |  |  |  |
| *Stachys palustris* |  | x |  |  |  |
| *Stachys sylvatica* | x | x |  | x |  |
| *Stellaria graminea* | x | x |  |  | x |
| *Stellaria media* |  | x |  |  |  |
| *Stellaria nemorum* |  | x |  |  |  |
| *Stellaria palustris* | x |  |  |  |  |
| *Succisa pratensis* | x | x |  |  | x |
| *Symphytum asperum* |  |  |  |  |  |
| *Symphytum uplandicum* |  | x |  |  |  |
| *Syringa vulgaris* |  | x |  |  |  |
| *Tanacetum macrophyllum* | x |  |  |  |  |
| *Tanacetum vulgare* | x | x |  | x |  |
| *Thalictrum flavum* | x |  |  |  |  |
| *Thalictrum simplex* | x |  | x |  | x |
| *Thlaspi arvense* |  | x |  |  |  |
| *Thlaspi caerulescens* |  | x |  |  |  |
| *Thymus serpyllum* | x | x |  |  | x |
| *Tilia cordata* |  |  |  |  |  |
| *Torilis japonica* | x | x |  |  |  |
| *Tragopogon pratensis* | x | x |  |  | x |
| *Trientalis europaea* |  |  |  |  |  |
| *Trifolium arvense* |  | x |  |  | x |
| *Trifolium aureum* |  | x |  |  | x |
| *Trifolium hybridum* | x | x |  |  |  |
| *Trifolium medium* | x |  |  |  |  |
| *Trifolium montanum* | x | x | x |  | x |
| *Trifolium pratense* | x | x |  |  | x |
| *Trifolium repens* | x | x |  | x |  |
| *Triglochin palustre* | x |  |  |  |  |
| *Tripleurospermum perforatum* |  | x |  |  |  |
| *Triticum aestivum* | x | x |  |  |  |
| *Tussilago farfara* | x | x |  | x |  |
| *Ulmus glabra* | x | x | x |  |  |
| *Urtica dioica* | x | x |  | x |  |
| *Vaccinium myrtillus* |  | x |  |  |  |
| *Vaccinium uliginosum* | x | x |  |  |  |
| *Vaccinium vitis-idaea* | x | x |  |  |  |
| *Valeriana sambucifolia* |  | x |  |  |  |
| *Verbascum nigrum* |  | x |  |  |  |
| *Verbascum thapsus* | x | x |  |  |  |
| *Veronica agrestis* |  | x |  |  |  |
| *Veronica arvensis* | x | x |  |  |  |
| *Veronica beccabunga* | x |  |  |  |  |
| *Veronica chamaedrys* | x | x |  |  | x |
| *Veronica officinalis* |  | x |  |  | x |
| *Veronica persica* |  | x |  |  |  |
| *Veronica scutellata* | x |  |  |  |  |
| *Veronica serpyllifolia* |  | x |  |  |  |
| *Veronica spicata* | x | x |  |  |  |
| *Viburnum opulus* | x | x |  |  |  |
| *Vicia cracca* |  | x |  |  |  |
| *Vicia hirsuta* | x | x |  |  |  |
| *Vicia sepium* | x | x |  |  |  |
| *Vicia sylvatica* |  | x |  |  |  |
| *Vicia tetrasperma* |  | x |  |  | x |
| *Vincetoxicum hirundinaria* | x | x |  |  |  |
| *Viola arvensis* | x | x |  |  |  |
| *Viola canina* |  | x |  |  | x |
| *Viola hirta* |  | x |  |  |  |
| *Viola mirabilis* |  | x |  |  |  |
| *Viola palustris* | x | x |  |  |  |
| *Viola riviniana* | x | x |  |  |  |
| *Viola tricolor* |  | x |  |  | x |
